# Muscle-Specific Promoters for Gene Therapy

**DOI:** 10.32607/actanaturae.11063

**Published:** 2021

**Authors:** V. V. Skopenkova, T. V. Egorova, M. V. Bardina

**Affiliations:** Institute of Gene Biology, Russian Academy of Sciences, Moscow, 119334 Russia; Marlin Biotech LLC, Moscow, 121205 Russia; Center for Precision Genome Editing and Genetic Technologies for Biomedicine, Institute of Gene Biology, Russian Academy of Sciences, Moscow, 119334 Russia

**Keywords:** Gene therapy, muscle-specific promoters, AAV, natural promoters, synthetic promoters

## Abstract

Many genetic diseases that are responsible for muscular disorders have been described to date. Gene replacement therapy is a state-of-the-art strategy used to treat such diseases. In this approach, the functional copy of a gene is delivered to the affected tissues using viral vectors. There is an urgent need for the design of short, regulatory sequences that would drive a high and robust expression of a therapeutic transgene in skeletal muscles, the diaphragm, and the heart, while exhibiting limited activity in non-target tissues. This review focuses on the development and improvement of muscle-specific promoters based on skeletal muscle α-actin, muscle creatine kinase, and desmin genes, as well as other genes expressed in muscles. The current approaches used to engineer synthetic muscle-specific promoters are described. Other elements of the viral vectors that contribute to tissue-specific expression are also discussed. A special feature of this review is the presence of up-to-date information on the clinical and preclinical trials of gene therapy drug candidates that utilize muscle-specific promoters.

## INTRODUCTION

Inherited muscle disorders are diagnosed in 4–5 people per 20,000 [1]. These diseases include the clinically and genetically heterogeneous group of muscular dystrophies, congenital myopathies, lysosomal storage disorders, channelopathies, and mitochondriopathies. Weakness of skeletal muscles limits locomotor activity, pharyngeal muscle dysfunction causes swallowing difficulties, while heart failure or respiratory insufficiency is a primary cause of early death. Unfortunately, effective treatment for such genetic muscle disorders does not exist [2].

**Table:** Inherited muscle disorders and the potential gene therapy

Disorder	Mutated gene	Inheritance pattern	Protein	Gene therapy drugs^*^ in clinical and preclinical studies
Duchenne muscular dystrophy Becker muscular dystrophy	DMD	XR	Dystrophin	CT:AAVrh74.MHCK7.miDMD NCT03769116 CT:AAV9.CK8e.miDMD, NCT03368742 CT: AAV9.tMCK.miDMD NCT04281485
Danon disease	LAMP2	XR	XR	PCT: AAV9.CAG.LAMP2B [4] CT: NCT03882437
Barth syndrome	TAZ	XR	Tafazzin	PCT: AAV9.Des.TAZ [5]
Myotubular myopathy	MTM1	XR	Myotubularin	PCT: AAV8.DES.hMTM1 [6] CT: NCT03199469
Primary merosin deficiency	LAMA2	AR	Merosin	PCT: AAV9.CB.mini-agrin [7]
Pompe disease	GAA	AR	α-1,4-Glucosidase	PCT: AAV2/8.MHCK7.hGAA [8] CT: AAV2/8.LSP.hGAA NCT03533673 CT: rAAV9.DES.hGAA NCT02240407
Limb-girdle muscular dystrophy LGMD, 2A	CAPN3	AR	Calpain 3	PCT: AAV9.desmin.hCAPN3 [9]
LGMD, 2B	DYSF	AR	Dysferlin	CT: rAAVrh.74.MHCK7.DYSF NCT02710500
LGMD, 2D	SGCA	AR	α-Sarcoglycan	CT: rAAV1.tMCK.hαSG NCT00494195 CT: scAAVrh74.tMCK.hSGCA NCT01976091
LGMD, 2E	SGCB	AR	β-Sarcoglycan	CT:scAAVrh74.MHCK7.hSGCB NCT03652259
LGMD, 2I	FKRP	AR	Fukutinrelated protein	PCT: AAV9.Des.mFkrp [10]
Oculopharyngeal muscular dystrophy	PABPN1	AD	PABPN1	PCT: AAV9.spc512.PABPN1 [11]

^*^Drug candidate name includes information about AAV serotype, promoter and transgene.

Note: AD – autosomal dominant; AR – autosomal recessive; XR – X-linked recessive; PCT – preclinical trials; CT – clinical trials.

Many inherited muscle diseases are caused by a protein deficiency resulting from mutations in the corresponding gene (*Table*). A promising strategy for treating such disorders is *gene replacement therapy, *which delivers a genetic construct with a functional copy of a gene (transgene) into muscle tissues. Adeno-associated virus (AAV) vectors are considered to be the most promising and safe for *in vivo *delivery of therapeutic genes [3]. Naturally occurring AAV serotypes such as AAV9, AAV8, AAVrh74, and AAV1 have an intrinsic tropism for muscles and allow for better targeting of affected tissues. Examples of AAV-based gene therapy candidates for inherited muscle disorders are listed in *Table*.

The therapeutic effect of gene therapy largely depends on the transgene expression levels in the targeting tissues. On one hand, muscles are a convenient target for gene therapy due to the long lifespan of muscle fibers, easy access for intramuscular injections, as well as high protein synthesis capacity [12]. On the other hand, muscles make up to 30–40% of body weight; so, high doses of the gene therapy drug are required [13]. Moreover, muscle tissue is structurally heterogeneous and is subdivided into cardiac, skeletal, and smooth muscles. This complicates the development of gene therapy drugs that would be equally effective in different types of muscle tissues [14].

A properly-selected promoter for transgene expression is the key to a successful gene therapy. This promoter should confer long-term sustained high expression in muscles affected by the disease, while exhibiting limited activity in other tissues. The popularity of AAV as a gene therapy vector makes necessary a reduction of the promoter size because of the limited packaging capacity of the virus (4.7 kbp) [3]. Strong constitutive promoters, such as the promoters of the respiratory syncytial virus (RSV), cytomegalovirus (CMV), or elongation factor 1a (EF1a), are compact in size and achieve high expression levels in a variety of tissues. However, it has been demonstrated that expression in non-target tissues, especially in antigenpresenting cells, triggers an immune response to the transgene and induces cytotoxicity [15]. Furthermore, viral promoters are prone to transcriptional silencing in transduced cells due to methylation [16].

This review focuses on strategies for designing and improving natural muscle-specific promoters. It highlights current approaches to the engineering of synthetic promoters, and it discusses their application in gene therapy constructs.

## THE STRUCTURE OF A EUKARYOTIC PROMOTER

**Fig. 1:**
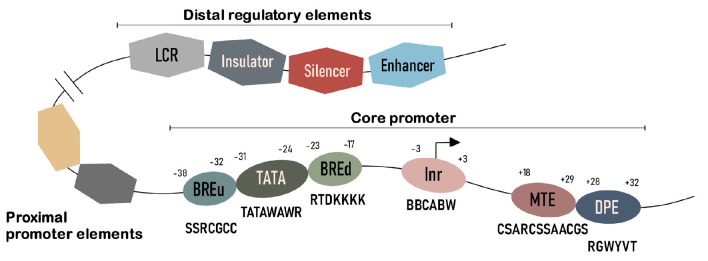
The structure of a eukaryotic promoter. The eukaryotic promoter consists of the core promoter, the proximal promoter elements, and distal regulatory elements. In the core promoter conserved motifs are shown with consensus sequences and the position from the transcription start site

Eukaryotic gene transcription is controlled by two classes of regulatory elements: promoters with core and proximal regions and distal regulatory elements (*Fig. 1*) [17].

The core (basal, or minimal) promoter is a specialized DNA sequence which directs transcription initiation and is located -50 to +50 bp from the transcription start site (TSS) [18]. There are several types of core promoters. The focused core promoter is a promoter with a single, well-defined TSS. The promoter with several closely positioned TSS within the 50–100 bp region is called dispersed [19]. The focused type is predominantly observed in promoters of tissue-specific genes, while dispersed core promoters are typical of universally expressed genes [18].

The core promoter supports the assembly of the preinitiation complex consisting of RNA polymerase II and basal transcription factors (TFs). Core promoters differ widely in terms of the conserved motifs that define their properties. Initiator (Inr) is the most common element of core promoters. The Inr sequence surrounds the TSS and is recognized by the multiprotein transcription factor II D (TFIID) [20]. Another well-characterized element of the core promoter is the TATA box. Approximately 28% of focused promoters in humans carry the TATA-like sequence [19]. This element is recognized by the TBP subunit of the transcription factor TFIID [21]. In promoters without the TATA box, Inr is often accompanied by the DPE motif (downstream promoter element), which is located downstream of the initiator and recognized by other subunits of TFIID [22]. The MTE (motif ten element) lies close to the DPE or overlaps with it [23]. Other typical elements of the core promoter include BREu (the upstream TFIIB recognition element) and BREd (the downstream TFIIB recognition element) [24].

The proximal promoter typically encompasses ~50–1,000 bp upstream of the TSS and contains many transcription factor binding sites (TFBSs) [25]. The unique combinations of these TFBSs in each promoter allow for tight regulation of the expression levels of ~25,000 human genes controlled by as few as ~1,600 TFs [26].

The distal regulatory elements of the eukaryotic gene include enhancers, silencers, insulators, and the locus-control regions (LCRs). Enhancer elements are of particular interest for promoter engineering. Enhancers are DNA sequences ~100- to 1,000-bp long that can increase the transcription of genes regardless of their orientation and distance to the target promoter [27]. These elements can be found in the 5’ and 3’ untranslated regions (UTRs) of the genes, within exons and introns, and even at a distance as large as 1 Mbp from the TSS [28]. Many enhancers are highly conserved sequences whose activity can be confined to a certain tissue or cell type, developmental stage, or certain physiological conditions [25].

## NATURAL PROMOTERS

A straightforward way to design a muscle-specific promoter is to use the naturally occurring promoter of the gene with high expression levels in muscles. To reduce the size of the full-length natural promoter, only the core promoter and some proximal elements are left and supplemented with distal enhancers [29]. Poorly conserved sequences are typically excluded from the design; the importance for the expression of the remaining promoter elements was verified by mutation analysis [29]. A similar approach is creating hybrid/ chimeric promoters by adding the enhancer elements of one gene to the promoter region of another gene [30]. The expression level and tissue specificity can be significantly improved by varying the copy number of the enhancers and individual TFBS and properly combining these sequences [31].

**Human skeletal α-actin promoters**

In their early attempts to create muscle-specific promoters, researchers focused on the promoter regions of proteins abundant in myocytes. Actin is the main protein that constitutes the sarcomere, the basic contractile unit of striated muscle. In higher vertebrates, six major isoforms of actin are distinguished, each encoded by a separate gene: skeletal and cardiac muscle α-actin, smooth muscle α-actin, smooth muscle γ-actin, and two isoforms with ubiquitous expression, cytoplasmic β-actin, and cytoplasmic γ-actin [32]. The human skeletal muscle α-actin gene (HSA) attracted the most interest from researchers, since this actin isoform prevails in adult muscles [33].

**Fig. 2:**
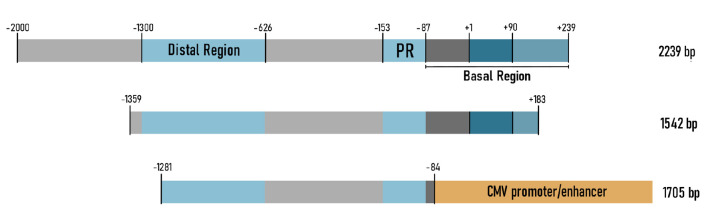
Promoters based on the *ACTA1/HSA *gene*. *(*A*) – the full-length HSA promoter includes the distal region, the proximal region (PR), and the basal region, which consists of the noncoding exon (+1...+90) and the first intron fragment (+91...+239); (*B*) – shortened version of the HSA promoter; (*C*) – the chimeric HSA/CMV promoter consisting of a fragment of the HSA promoter and the CMV promoter

The first studies demonstrated that the region located 2,000 bp upstream of the *HSA *gene, as well as the first exon and the fragment of the first intron, is necessary and sufficient for muscle-specific expression in a cell culture (*Fig. 2A*) [34]. Three major promoter regions have been identified: the distal (from -1300 to -626 from the TSS), the proximal (-153...-87), and the basal   (-87...+239) regions. These promoter regions, lumped together or separately before the SV40 promoter, drive tissue-specific expression [34].

The fragment of *HSA *gene extending from c -2,000 bp to +7,500 bp (promoter region -2,000...+239 as in *Fig. 2A*) was used to generate a transgenic mouse line in [35]. It was demonstrated for the first time that the transgene expression was comparable to the expression level of endogenous mouse skeletal muscle α-actin in the striated muscles and the heart. The HSA promoter has become rather popular and has been used in a number of studies. For example, it was employed to produce transgenic mice carrying dystrophin gene deletions [36], mice with dysferlin overexpression [37], and a mouse model of spinal muscular atrophy [38], as well as to deliver microdystrophin into mouse muscles using lentiviral vectors [39].

Another truncated variant of the human HSA promoter was used in the AAV vector to treat Duchenne muscular dystrophy [40]. A high expression level in the muscles was achieved using a 1,542-bp fragment consisting of a distal region, a promoter, and a portion of the first intron (*Fig. 2B*). The transgene was actively expressed in skeletal muscles and the heart, but no transgene expression was detected in the liver.

The chimeric HSA promoter was used to produce coagulation factor IX in muscles and treat hemophilia B [41]. This promoter was a fragment of the HSA promoter (-1281...-84) ligated to the CMV promoter (*Fig. 2C*). In the myoblast cell culture, the transgene expression level ensured by this promoter was higher than the transgene expression levels induced by the CMV promoter and the full-length HSA promoter, while this promoter was as active as the CMV promoter in nonmuscle cell cultures. It appears that, although the addition of the universally expressed CMV promoter increased the activity of the chimeric promoter, it became tissue non-specific.

The regulatory regions of the homologous chicken [42], rat [43], and bovine [44] genes were modified to design muscle-specific promoters similar to the HSA one. The resulting constructs have been successfully used in *in vitro *and *in vivo *experiments, as well as to generate transgenic mice.

In general, the skeletal muscle α-actin promoters exhibited a high expression level and specificity in muscle cells; however, they are used in modern studies not so frequently, because of their large size.

**Muscle creatine kinase promoters**

The transcript of the muscle creatine kinase (*MCK/CKM, *creatine kinase, M-type) gene is the second-most abundant mRNA in skeletal muscles [45]. MCK catalyzes reversible phosphoryl transfer from ATP to creatine and from creatine phosphate to ADP, thus providing energy for muscle contractions. The* MCK *gene is also highly active in the cardiac muscle and is transcriptionally activated during the differentiation of myoblasts into myocytes [46].

The *MCK *promoter has been characterized well both *in vitro *and *in vivo*. One of the major regulatory regions of the mouse *Mck *gene is the muscle-specific 206-bp enhancer located within the -1256...-1050 region [47]. This enhancer exerts its function regardless of orientation and carries a number of binding sites for myogenic transcription factors (namely, E-boxes, CArG, and MEF2 sites). A mutation analysis of these motifs has confirmed their importance in muscle-specific expression [48].

The proximal promoter (358 bp) is the key regulatory element of the *MCK *gene [47]. As such, it alone ensured a high expression level of the transgene in limb muscles and abdominal skeletal muscles in mice but was inactive in cardiac and tongue muscles. Nevertheless, when the 206-bp enhancer and the 358-bp promoter were ligated together, expression was restored Fig. 2. Promoters based on the *ACTA1/HSA *gene*. *(*A*) – the full-length HSA promoter includes the distal region, the proximal region (PR), and the basal region, which consists of the noncoding exon (+1...+90) and the first intron fragment (+91...+239); (*B*) – shortened version of the HSA promoter; (*C*) – the chimeric HSA/CMV promoter consisting of a fragment of the HSA promoter and the CMV promoter in all types of muscles [47]. The data obtained for transgenic mice have proved that the enhancer is required in order to induce expression in the heart [49].

**Fig. 3:**
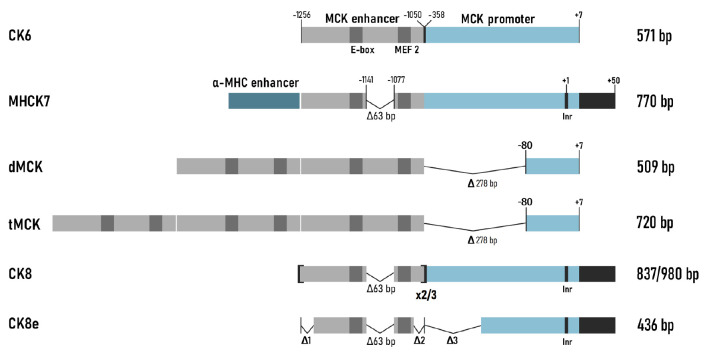
*Mck*-based promoters. All the constructs contain the MCK enhancer and the MCK promoter, with different modifications

Based on the discussed-above regulatory sequences, several series of small MCK expression cassettes for adenoviral vectors were developed and tested [50]. Thus, the **construct CK6, **consisting of an enhancer (206 bp) and a proximal promoter (358 bp) (*Fig. 3*), ensured high muscle specificity. However, the expression level in the muscles was ~12% compared to that attained using a similar construct with the CMV promoter; the expression level in the heart remained low [50].

The chimeric promoter **MHCK7 **was developed to achieve a high expression level of the transgene in the cardiac muscle (*Fig. 3*) [29]. It included a 206-bp enhancer and a proximal promoter but contained four important modifications. Thus, the poorly conserved region between the right E-box and the MEF2 site was deleted in the 206-bp enhancer. The highly conserved 50-bp sequence from the first noncoding exon of *MCK* was added to the promoter. The TSS-containing sequence was replaced with the Inr consensus sequence. The most important modification was the following: a 188-bp enhancer from the mouse α-myosin heavy chain gene (α-*Mhc*), which ensures a high expression level in the heart, was added to the expression cassette described above [51]. The new MHCK7 promoter was tested in the context of AAV vectors. The promoter ensured a transgene expression level comparable to those for the CMV and RSV promoters in skeletal and cardiac muscles. Low expression levels were observed in the liver, lungs, and spleen after AAV6 had been intravenously injected to mice. Interestingly, the MHCK7 promoter was 400 and 50 times more active in the heart and the diaphragm, respectively, than promoter CK6.

The **dMCK **and **tMCK **promoters (*Fig. 3*) were developed in another laboratory, almost simultaneously with the MHCK7 promoter [52]. In these constructs, the proximal promoter (358 bp) was shortened to a 87-bp basal promoter (-80...+7) and two or three copies of the MCK enhancer (206 bp) were ligated to it. In the experiments where the transgene was delivered using AAV vectors, tMCK proved to be the most efficient promoter; the level of muscle-specific expression it ensured was higher than the expression levels ensured by the CMV, dMCK, and CK6 promoters. However, the dMCK promoter did not activate transgene expression in the heart or diaphragm.

However, the search for efficient muscle-specific promoters continued. The constructs named **CK8** (*Fig. 3*) were developed at the very same laboratory where the CK6 and MHCK7 promoters had been previously created. Thus, the CK8 promoter (MHCK7 with two copies of the MCK enhancer instead of the α-myosin heavy-chain enhancer) was used for intramuscular AAV8-mediated delivery of the growth hormone gene [53]. In mice treated with this construct, body length and weight were significantly higher compared to those in untreated mice. In another study, the CK8 promoter was similar to the construct described above but carried three copies of the MCK enhancer [31]. It was reported that using three instead of two enhancer copies increases the expression levels in skeletal myocytes and in the heart four- and threefold, respectively [31]. The 436-bp **CK8e **construct carried a number of deletions in the enhancer and proximal promoter of muscle-type creatine kinase (*Fig. 3*) and was more active than the CMV promoter in a differentiated human myoblast culture [54]. Deletion of the poorly conserved regions in the promoter reduced its length and simultaneously increased its activity [31].

Due to a high specificity and activity in muscle tissues, promoters based on the *MCK *gene are widely used in the gene therapy vectors that are currently undergoing preclinical and clinical testing (*Table*). The MHCK7 promoter is included in a vector undergoing preclinical studies for the treatment of the Pompe disease [8]. Clinical trials to treat LGMD type E (NCT03652259), where a functional copy of the β-sarcoglycan is delivered into patients under the control of the MHCK7 promoter, are currently underway [55]. The MHCK7 promoter is also being used as part of a construct to deliver the dysferlin gene (NCT02710500) [56]. In the ongoing clinical studies (NCT03769116) focused on the treatment of Duchenne muscular dystrophy the microdystrophin gene is delivered into patients under the control of the MHCK7 promoter [57]. The CK8e promoter was used in a clinical trial focusing on microdystrophin delivery using AAV9 NCT03368742) in [58]; another clinical study (NCT04281485) focused on the tMCK promoter and AAV9-mediated delivery of the microdystrophin gene [57]. A clinical trial (NCT01976091) evaluating the delivery of the α-sarcoglycan gene under the control of the tMCK promoter in the treatment of LGMD type 2D is also underway.

**Desmin gene promoters**

Desmin is a muscle-specific cytoskeletal protein belonging to the intermediate filament family [59]. It is encoded by the *DES *gene and is one of the earliest myogenic markers [59]. This protein is unique in that it is expressed in satellite cells and dividing myoblasts, while its abundance in differentiated muscle cells is several times higher [60].

A functional analysis of the 5’-flanking region of the human desmin gene revealed an enhancer (-973...-693) [60]. The 5’-region of the enhancer contains the MEF2-binding sites, the E-box, and the Mt element; it is needed for activating expression in muscle fibers. The 3’-half of the enhancer is responsible for desmin transcription in myoblasts because of binding to SP1 and KROX-20 [60]. The region -692...-228 is a silencer, which reduces expression in myoblasts and muscle fibers by up to 3- to 7-fold; the region -228...+75 was sufficient to initiate a low-level muscle-specific expression [61, 62].

**Fig. 4:**
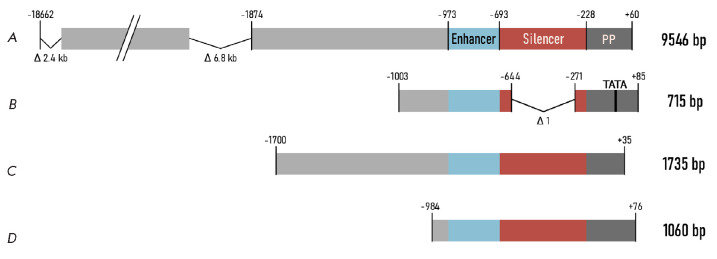
Promoters based on the human *DES *gene. Promoter (*A*) includes the locus control region of the desmin gene (18.7 kbp) with introduced deletions, the enhancer, the silencer, and the proximal promoter (PP). Promoter (*B*) contains a deletion in the silencer and the TATA box added to the core promoter. Promoters (*C*) and (*D*) have deletions in the distal regions

The full-length dystrophin gene under the control of the human desmin promoter (9546 bp; region -18662...+60) in a plasmid vector was used for intraarterial delivery in mice (*Fig. 4A*) [63]. This promoter ensured the same level of dystrophin expression as the CMV promoter did for at least 6 months.

A variant of the human desmin promoter (715 bp) with a deleted silencer was used in a comparative study of muscle-specific promoters for intravenous AAV9- mediated transgene delivery (*Fig. 4B*) [64]. In that study, the desmin promoter was superior to the CMV promoter and other muscle-specific promoters in terms of the transgene expression level attained in skeletal muscles and the diaphragm. In terms of the expression level in the heart, it was less effective only than the CMV promoter, while it still ensured a high level of transgene expression in the brain [64].

Another variant of the human desmin promoter was used in a study focused on transgene delivery in mouse muscles using lentiviral vectors [65]. The region (-1700...+35) containing a promoter, a silencer, and an enhancer was used (*Fig. 4C*). The activity of this promoter was comparable to that of the CMV promoter in experiments *in vitro *and *in vivo*, being even higher than that of the human *MCK *promoter (-1061...+28).

Desmin promoters were used in a number of studies (*Table*) focused on the Pompe disease [66]. The construct rAAV9.DES.hGAA, intended to treat this disorder, is so far successfully undergoing clinical trials (NCT02240407). A promoter variant [64] was used in preclinical studies to develop gene therapy drugs for patients with the Barth syndrome [5]. Furthermore, the desmin promoter was used in preclinical studies as a vector to treat LGMD type 2A (calpain 3 deficiency) [9] and LGMD type 2I (fukutin-related protein deficiency) [10]. The human desmin promoter (-984...+76) (*Fig. 4D*) was evaluated in preclinical studies focused on the therapy of myotubular myopathy, a genetic disorder caused by mutations in the *MTM1 *gene [6]; this medicinal product has almost completed clinical trials (NCT03199469).

**Promoters based on other genes**

The regulatory regions of many other genes that exhibit muscle-specific expression were also used to construct promoters. A search for a candidate promoter was simultaneously conducted in a number of laboratories, but only a few studies proved successful.

For instance, to treat the cardiac variant of the Fabry disease, lentiviral constructs with cardiac-specific transgene expression were developed [67]. Three different promoters were tested: the human α-myosin heavy chain gene (αMHC) promoter (region -1198...+1), the myosin light-chain promoter (**MLC2v**) (-250...+13), and the cardiac troponin T promoter (**cTnT**) (-300...+1). All three promoters were superior to the ubiquitous EF1α promoter in their expression levels of transgene in the heart. Besides the cardiac expression, the cTnT and MLC2v promoters also drove expression in the liver and spleen, while the transgene, under the control of αMHC, was active exclusively in the heart [67]. However, another study revealed that promoters based on these genes were inferior to the desmin promoter in terms of their expression level in skeletal muscles and the heart [64].

A chimeric promoter comprising the CMV-IE enhancer ligated to the 1.5-kbp fragment of the rat promoter** MLC **was used for AAV9-mediated delivery of microdystrophin in a mouse heart [68]. The cardiac activity of this promoter was four times as high as that of the CMV, and robust transgene expression in the heart was conferred for 10 months, but not in the skeletal muscles or the liver [69].

The ΔUSEx3 promoter was developed on the basis of the human troponin I (*TNNI1*) gene and consisted of three copies of the enhancer (-1036...-873) and the minimal promoter of the *TNNI1 *gene with a portion of the first exon (-95...+56) [70]. The ΔUSEx3 promoter exhibited weak activity in nonmuscle cells and tissues in *in vivo *and *in vitro *experiments. Let us mention that the ΔUSEx3 promoter delivered by adenoviruses ensured a transgene expression level comparable to that induced by the synthetic SPcΔ5-12 promoter [71]; however, ΔUSEx3 delivered by lentiviruses was five times less active than the SPcΔ5-12 promoter, probably due to the effects related to its integration into the genome.

The unc45b gene encoding the muscle-specific myosin chaperone in fish was also used to develop a muscle-specific promoter [72]. Thus, the 195-bp promoter fragment (-505...-310) was able to induce expression in skeletal and cardiac muscles in fish and ensured reporter protein expression in mouse muscles when the plasmids were delivered by electroporation.

To summarize, the promoters discussed in this section proved capable of driving muscle-specific expression but were less potent than the promoters based on the actin, muscle creatine kinase, or desmin genes.

**Synthetic promoters**

A groundbreaking approach in promoter design is the creation of novel *synthetic promoters*. This strategy enables one to engineer promoters with defined properties, such as size and the expression profile of the transgene.

The development of synthetic promoters relies on computational algorithms, which are used to identify regulatory sequences and TFBSs within the genome, as well as to predict the promoter regions [73, 74, 75]. The binding sites for myogenic TFs are usually shorter than 10 bp [74], which allows one to create a library of constructs with different combinations of muscle-specific TFBSs. The key challenge in this approach is to analyze large libraries of novel synthetic constructs, which can be labor-intensive. Experiments are needed in order to determine the number of copies of the target motif and the distances between TFBS required for a successful binding of the transcription factor; not to mention identify the motifs having a synergistic function and the TFBS making the greatest contribution to expression enhancement. In order to overcome these obstacles, one can return to the analysis of natural promoters: extract the functioning combinations of muscle-specific TFBS and construct promoters from similar clusters. An *in silico *analysis can substantially simplify the detection of regulatory regions.

**Fig. 5:**
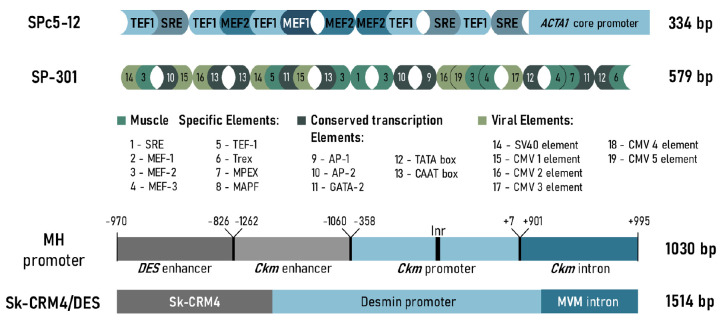
promoters. The SPc5-12 promoter consists of a combination of four muscle-specific TFBSs (TEF1, SRE, MEF1, and MEF2) and the core promoter (a fragment of the promoter of the chicken skeletal muscle α-actin gene). The SP-301 promoter is a combination of muscle-specific TFBSs, viral elements, and conserved *cis*-regulatory elements ligated in forward and reverse orientation. The MH promoter consists of the human desmin gene enhancer linked to the enhancer, the core promoter, and the first intron of the mouse *Ckm *gene. Sk-CRM4/Des is the regulatory module Sk-CRM4 ligated to the desmin promoter and the MVM intron

In their pioneering study, Li *et al*. analyzed the sequences of strong muscle-specific promoters and identified the common binding sites of myogenic TFs (SRE, MEF-2, MEF-1, and TEF-1) within their structure [71]. These TFs were randomly ligated to each other in forward and reverse orientation, and the resulting fragments were inserted upstream of the minimal promoter of the chicken skeletal muscle α-actin gene (*Fig. 5*). As a result, a library consisting of more than 1,000 promoter variants was created. The synthetic promoter library was screened in primary myoblasts, and, based on the results, the **SPc5-12 **promoter was selected (*Fig. 5*). SPc5-12 activity in muscle fibers was sixfold higher than that of the CMV promoter. The *in vivo *experiments confirmed that the SPc5-12 promoter is inactive in undifferentiated myoblasts and in various nonmuscle cell lines.

The SPc5-12 promoter was used to drive transgene expression in animal models of Duchenne muscular dystrophy [76], the Pompe disease [77], and dysferlinopathy [78], as well as to ensure growth hormone expression [79]. A gene therapy construct with the SPc5-12 promoter was utilized in preclinical studies for the treatment of oculopharyngeal muscular dystrophy [11].

Liu *et al. *used a similar strategy to construct synthetic promoters [80]. The promoters were designed from 19 elements, including eight muscle-specific TFBS, six viral elements (CMV and sv40 promoters), and five conserved *cis*-regulatory elements of eukaryotic promoters (TATA box, etc.). These motifs were randomly assembled to construct a library consisting of 1,200 primary clones, which were tested *in vitro* and *in vivo*. The strongest transcriptional activity was achieved with **the SP-301 **promoter (*Fig. 5*); it was 6.6 times more active than the CMV promoter 2 days after intramuscular delivery of the construct in mice and remained active for at least a month. Many promoters achieved a higher *in vitro *activity compared to the CMV promoter, but they were less active *in vivo*. The tissue specificity of the SP-301 promoter was confirmed in transgenic mice. This study once again demonstrated the advantage of the strategy of designing synthetic promoters using a combination of TFBSs and also highlighted the the benefit of including viral motifs besides muscle-specific TFBSs for enhanced expression levels.

Another efficient approach, consisting in designing hybrid promoters, has already been partially discussed for the MHCK7 promoter [29]. In the study where this strategy was employed [30], Piekarowicz* et al*. conducted an *in silico *analysis of various tissuespecific genes and identified four clusters that con- sisted of a combination of the binding sites of myogenic transcription factors. The first cluster was the previously discussed enhancer of the human desmin gene (-970...-826) [81]; the remaining three clusters were regions of the enhancer (-1256...-1051), the proximal promoter (-358...+7), and the first intron (+901...+995) of the mouse *Ckm *gene. The promoter that contained all four elements (the **MH promoter**) ensured the highest expression level in the muscle cell culture, being superior to the desmin and CMV promoters, as well as the remaining hybrid promoters. Interestingly, intron made the greatest contribution to the expression level, while deletion of one or the two enhancers or even the core promoter did not significantly alter the expression level. To test the *in vivo *activity of the hybrid promoter, AAV2/9 carrying the reporter gene was delivered intravenously in mice under the control of this promoter. The activity of the MH promoter in the cardiac and skeletal muscles was higher than that of the desmin and CMV promoters; however, the MH promoter did not induce transgene expression in the liver [30].

The strategy of using muscle-specific *cis*-regulatory modules (**Sk-CRM**) was employed in the next study [82]. TFBSs were mapped in the promoters of human genes highly expressed in skeletal muscles and analyzed for their tendency to form clusters. To identify conserved motifs, these clusters were subjected to multiplesequence alignment across various animal species. It was assumed that the TFBS combinations conserved in evolution are more likely to retain potency and specificity following clinical translation. Open chromatin structure and the accessibility of candidate Sk-CRMs to TFs were also taken into consideration. Using this computational approach, seven novel evolutionarily conserved muscle-specific Sk-CRMs modules were identified and cloned upstream of the desmin promoter. Based on the results of a bioluminescence assay, the** Sk-CRM4 **module was selected for further studies (*Fig. 5*). Six weeks after systemic delivery using AAV9, the Sk-CRM4 chimeric promoter enhanced the activity of the desmin promoter by 200–400 times in different skeletal muscles, the diaphragm, and the heart, while remaining inactive in non-target tissues. Moreover, the SkCRM4/Des promoter attained a 25–173 times higher expression in different muscles as compared to the CMV promoter and also outperformed the Sk-CRM4/ SPc5-12 and SPc5-12 promoters. Therefore, the computationally designed Sk-CRM4/Des chimeric promoter demonstrated improved muscle-specific performance as compared to the other promoters commonly used for muscle gene therapy, with length (~1,500 bp) being its only drawback.

To conclude, implication of synthetic promoters in gene therapy holds great promise. Success in synthetic promoter engineering largely depends on the quality of the bioinformatic tools and efficient screening of the proposed variants. Expansion of the regulatory element databases, revealing new TFs, and improvement in the software for promoter identification will undoubtedly contribute to further development in this area of research [73, 74, 75].

## OTHER FACTORS DETERMINING MUSCLE-SPECIFIC EXPRESSION

When developing gene therapy drugs, one should comprehensively evaluate the expression of the genes of interest at the proper position and time, as this depends not only on promoter activity, but on other factors as well. Many factors affect the transgene expression at the post-transcriptional level.

**Fig. 6:**
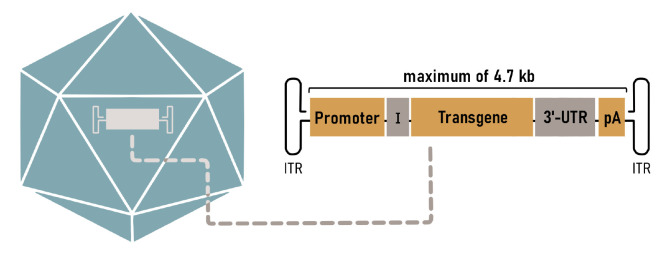
Typical elements of the AVV expression cassette. Orange blocks (the promoter, the transgene, and polyadenylation signal (pA)) are the basic components of the cassette. Accessory *cis*-regulatory elements, such as intron (I), WPRE, and the microRNA binding sites (3’-UTR), can also be inserted to enhance expression efficiency. The cassette is flanked by inverted terminal repeats (ITR)

Expression of the target gene can be enhanced due to the presence of an intron in the vector, which is usually positioned between the promoter and the coding region (*Fig. 6*). The presence of the intron increases RNA stability in the nucleus due to the incorporation of mRNA into the spliceosome [74] and promotes efficient export of spliced mRNA from the nucleus to the cytoplasm [83]. Introns can also contain regulatory sequences that affect tissue specificity and the expression level. A study focused on designing a chimeric promoter [30] showed that the presence of intron from the *Ckm *gene makes the greatest contribution to the transgene expression level. The MVM intron enhanced transgene expression during an AAV-mediated delivery of coagulation factor IX more than 80-fold compared to the construct without intron [84].

Along with the promoters, other *cis*-regulatory elements can also be added to the 3’-UTR of the expression cassette to enhance expression (*Fig. 6*). Thus, the 600-bp post-transcriptional regulatory element of the woodchuck hepatitis virus (WPRE) delivered using AAV led to a manifold enhancement of transgene expression in the liver, brain, and muscles [85]. WPRE promotes mRNA export from the nucleus and prevents post-translational gene silencing [86].

A different approach can also be used to achieve tissue specificity: not only inducing expression in the target tissues, but also suppressing it in non-target organs through RNA interference mechanisms [74]. For this purpose, the binding sites of microRNA that are present only in the non-target organs are added to the 3’-UTR of the expression cassette (*Fig. 6*) [87]. If transgenic mRNA is expressed in a non-target organ, microRNA binds to the complementary sites on the transgene and initiates its degradation [87].

A proper choice of viral vector also plays a significant role in the delivery of the transgene into the target organs and tissues. Along with the naturally occurring serotypes of AAVs (*Table*), capsids are also modified to design novel, genetically engineered vectors and improve the targeted delivery [88]. There is an ongoing search for other naturally occurring capsids with improved tropism for the heart and skeletal muscles [89]. Transgene expression patterns also differ depending on the route of administration (intravenous, intramuscular, etc*.*) [90]. An elaborate combination of the above-mentioned elements in the cassette, proper choice of the viral vector, and an optimal delivery route for the genetically engineered drug can significantly enhance the expression of the gene of interest, while maintaining tissue-specific expression.

## CONCLUSIONS

The efforts to develop optimal muscle-specific promoters started more than 30 years ago and are still underway. Early versions of natural muscle-specific promoters consisted of the regulatory regions of the actin, desmin, and muscle creatine kinase genes and exceeded 1 kbp in length. The latest generations of synthetic promoters contain combinations of TFBS from common muscle-specific genes, are much shorter, but the expression efficiency achieved by these promoters is comparable to or higher than that of natural promoters [30, 71].

It has been demonstrated in many studies that transcription factors and their binding sites in vertebrates are appreciably conserved [42, 72]. Thanks to this property of promoters, various animal models can be used in preclinical studies to prove the effectiveness of gene therapy drugs. However, when conducting *in vitro *studies, one should keep in mind that promoter activity in this case does not necessarily coincide with* in vivo *activity [80].

Since the group of genetic muscular disorders is heterogeneous, there is no universal promoter that could be used to develop vectors intended for the treatment of all diseases. This can be largely attributed to the features of the pathogenesis and the different functions of the proteins whose deficiency or dysfunction causes a given disorder *(Table)*. Different muscle groups and types of muscle fibers are affected in patients with different disorders [14]. The gained experience in developing muscle-specific synthetic promoters provides hope that researchers will eventually design ideal constructs that mimic the unique expression profile of musclespecific proteins and fully restore their lost functions.

## Abbreviations

AAV: adeno-associated virus
PCT: preclinical trials
CT: clinical trials
LGMD: limb-girdle muscular dystrophy
UTR: untranslated region
TF: transcription factor
CMV: cytomegalovirus
MVM: minute virus of mice
TSS: transcription start site
TFBS: transcription factor binding site
